# Biomarkers and Bioassays for Cardiovascular Diseases: Present and Future

**Published:** 2008-05-19

**Authors:** Derek S. Sim, Hsiao Lieu, Patrick Andre

**Affiliations:** 1 Department of Biology; 2 Department of Clinical and Regulatory Affairs, Portola Pharmaceuticals Inc., South San Francisco, CA

**Keywords:** troponin, CK-MB, clopidogrel, aspirin, perfusion chamber

## Abstract

Stratification of cardiac patients arriving at the emergency department is now being made according to the levels of acute cardiac biomarkers (i.e. cardiac troponin (cTn) or creatine kinase myocardial band (CK-MB)). Ongoing efforts are undertaken in an attempt to identify and validate additional cardiac biomarkers, for example, interleukin-6, soluble CD40L, and C-reactive protein, in order to further risk stratify patients with acute coronary syndrome. Several studies have also now shown an association of platelet transcriptome and genomic single nucleotide polymorphisms with myocardial infarction by using advanced genomic tools. A number of markers, such as myeloid-related protein 14 (MRP-14), cyclooxygenase-1 (COX-1), 5-lipoxygenase activating protein (FLAP), leukotriene A_4_ hydrolase (LTA4H) and myocyte enhancing factor 2A (MEF2A), have been linked to acute coronary syndromes, including myocardial infarction. In the future, these novel markers may pave the way toward personalized disease-prevention programs based on a person’s genomic, thrombotic and cardiovascular profiles. Current and future biomarkers and bioassays for identifying at-risk patients will be discussed in this review.

## Introduction

Cardiovascular disease claims over 10 million lives annually worldwide. The development of cardiac biomarkers has allowed physicians to make life-saving treatment decisions in a safe and cost-effective manner by reducing overly-aggressive therapy regimens and nonessential invasive procedures. Currently, when cardiovascular patients arrive to the emergency department, treatment decisions are guided by the medical history, physical examination, electrocardiogram results, and the levels of cardiac-specific biomarkers. Due to the sensitivity and specificity of cardiac biomarkers, one can rapidly determine the level of myocardial necrosis and the risk level of a patient. Plasma biomarkers such as troponin and creatine kinase myocardial band (CK-MB) are the frontline markers aiding clinicians in the diagnosis of cardiac ischemia. While these cardiac enzymes are invaluable in acute clinical settings for directing treatment, their presence only represents a consequence of cardiac events. Recent advances in genomics open up new avenues for identifying genetic risk factors decades before patients develop heart disease. Although it is likely to take many more years to develop and validate these genetic markers, the successful development of inhibitors to these susceptible genes and gene products would provide the prospect of interfering the progression of cardiovascular disease early in development and reduce clinical events.

In addition to early identification of cardiac disease, the monitoring of the response to therapy is also crucial in terms of cardiac patient management. For example, the acute biomarker levels, such as troponin and CK-MB, provide risk stratification and direct indication of the effectiveness of the treatment and the long term prognosis. High levels of these acute cardiac biomarkers are usually associated with poor clinical outcome (Lincoff, Bittl et al. 2003; Kastrati, Mehilli et al. 2006). Due to a heterogeneous response of cardiac patients to antiplatelet therapies, bioassays are being developed and used to measure the level of platelet inhibition to directly evaluate the level of protection these patients are receiving from the anti-platelet therapy and to identify patients who would require a more aggressive dosage. In this review, some of the current and future biomarkers for identifying at-risk patients and bioassays for evaluating the efficacy and possibly individual tailoring of anti-platelet therapies will be discussed.

## Biomarkers for Cardiovascular Diseases

### Acute biomarkers for diagnosing cardiac events

The development of acute cardiac markers has provided a way for physicians to promptly determine the level of myocardial necrosis a patient has suffered. This information helps clinicians to make decisions on the use of appropriate antithrombotic therapies and interventions for patients diagnosed with ACS. The biomarkers for myocardial necrosis include creatine kinase-myocardial band (CK-MB) and cardiac troponin (cTN).

#### Creatine kinase-myocardial band (CK-MB)

CK-MB has been a predominant marker for identifying ACS patients with myocardial necrosis. Circulating CK-MB originates from both cardiac and skeletal muscle sources and therefore is not entirely cardiospecific (Panteghini, 1995). Historically, CK-MB played an important role for years in aiding ACS diagnosis, detecting myocardial injury, and prognosis. A large number of studies have been published to support the diagnostic and prognostic values of CK-MB in acute coronary syndrome (ACS) patients. However, recent large registries are showing that troponin is more sensitive and associated with increased risk even in CK-MB negative ACS patients. In the CRUSADE initiative comparing troponin and CK-MB (Newby, Roe et al. 2006), there was a discordant result in 28% of NSTEMI patients. While 18% of the patients had elevated troponin but normal CK-MB values, 10% had false-positive CK-MB, as defined by normal troponin values. Compared to patients who were negative for both markers, troponin-positive/CK-MB-negative patients had a trend toward increased mortality (4.5% versus 2.7%). The differences in outcomes could not be explained by differences in therapy. Thus, an isolated troponin elevation in the setting of negative CK-MB was associated with increased risk. A similar finding has been noted in over 10,000 ACS patients in the GRACE registry (Goodman, Steg et al. 2006). For these reasons, cardiac troponin (cTn) is currently the preferred assay and more commonly used nowadays (Jaffe, Ravkilde et al. 2000).

#### Troponin

Cardiac troponin (cTn) is currently the preferred biomarker for the diagnosis and prognosis of acute cardiac diseases. Troponin is a cytoskeletal protein that consists of three subunits, troponin T (TnT), troponin I (TnI) and troponin C (TnC) (Filatov, Katrukha et al. 1999). These subunits are present in both skeletal muscle and cardiac muscle as different isoforms. Cardiac-specific isoforms of TnT (Anderson, Malouf et al. 1991) and TnI (Cummins and Perry, 1978) have been identified. Their tissue specificities allow them being highly specific and sensitive biomarkers for myocardial injury. In contrast, the cardiac isoform of TnC is present in both skeletal and cardiac muscles. Non-cardiac muscle myopathies can lead to an increase in plasma TnC. Therefore, TnC is not used as a specific marker of cardiac injury (Schreier, Kedes et al. 1990). Cardiac Tn is present as a cytosolic pool and a structural pool. Blood level of cTn increases as early as 1 to 10 hours after the onset of cardiac injury (Mair, Artner-Dworzak et al. 1991; de Winter, Koster et al. 1995). The initial release of cTn originates from the cytosolic pool and the subsequent release is from the structural pool. This increase in plasma cTnT and cTnI levels can be detected by current immunoassays. Current assays can detect cTn at levels from 0.01 μg/L to 1 μg/L. The European Society of Cardiology/American College of Cardiology task force has defined the upper limit of normal troponin level as the level above the 99th percentile in a normal population. For making clinical decisions, the troponin level is generally used together with 12-lead electrocardiogram (ECG) results to stratify patients into risk groups when they are being admitted to the hospital (Savonitto, Ardissino et al. 1999; Braunwald, Antman et al. 2002). Troponin level elevation also confers an independent prognostic purpose. Elevation of cTn in ACS patients was correlated with increased mortality at 42 days (Antman, Tanasijevic et al. 1996).

Most of the patients with ACS do not have ST-elevation. In these patients, elevated cTn identifies the subgroup of non-ST elevation myocardial infarction (NSTEMI) patients, which are at increased risk compared to cTN-negative individuals (Dokainish, Pillai et al. 2005; Ohtani, Ueda et al. 2005; Thom, Haase et al. 2006). Thus, it is important to initiate therapy as soon as the diagnosis has been made. For this subgroup of NSTEMI patients positive in troponin, the use of enoxaparin in combination with aspirin has been shown to reduce the risk of death, myocardial infarction or urgent target vessel revascularization by 47% within 14 days (Morrow, Antman et al. 2000). These cTn-positive NSTEMI patients also respond favorably to GPIIb/IIIa antagonists (Newby, Ohman et al. 2001) and clopidogrel (Yusuf, Zhao et al. 2001). Early invasive interventional strategy is also effective in significantly reducing clinical events (Cannon, 2003).

Patients with ST-elevation on ECG and a clinical history suggesting myocardial ischemia and a duration of symptoms less than 12 hours should receive immediate treatment regardless of cTN level. This is because ECG signs for cardiac ischemia occur before troponin elevation. Levels of troponin elevation in ST-elevation myocardial infarction (STEMI) patients had been associated with increased risk of short term and long term major adverse cardiac events and death (Ohman, Armstrong et al. 1996; Stubbs, Collinson et al. 1996; Gertz, Fallon et al. 1998; Ohman, Armstrong et al. 1999; Matetzky, Sharir et al. 2000). Delay in seeking medical help after the onset of symptoms results in more myocardial damage and elevation of troponin level. Studies have shown that patients admitted with positive troponin and ST-elevation have significantly higher mortality rate than patients negative in troponin, which reflects the adverse effect of delaying treatment. In one of the studies, STEMI patients with elevated cTnT (≥0.1 μg/L) had a mortality rate of 12.5% at 30 days and 14% at 9 months versus 3.9% and 3.9% respectively in patients negative of cTnT (Giannitsis, Muller-Bardorff et al. 2001). This group of high risk ACS patients should immediately undergo an early invasive treatment regimen.

For patients who have undergone percutaneous coronary intervention (PCI), a number of studies reported that the elevation of post-PCI cTn is associated with increased risk of adverse events (Morrow, Cannon et al. 2001; Cantor, Newby et al. 2002; Kizer, Muttrej et al. 2003; Nageh, Sherwood et al. 2003). However, a recent study has suggested that it is the baseline pre-PCI cTn rather than the post-PCI cTn level that is predictive of adverse events. They argue that for studies that showed elevated cTn post-procedure to be associated with adverse events did not take the pre-PCI cTn level into consideration when establishing the correlation (Miller, Garratt et al. 2006). In their study of 2352 patients with progressive symptomatic coronary artery disease or acute coronary syndrome, 11.1% patients with pre-PCI cTn elevation (≥0.3 μg/L) suffered from adverse events within a year in comparison to only 4.7% of patients without pre-PCI cTn elevation and post-PCI cTn level was not predictive of adverse events when baseline elevation was included in the analysis. Yet for patients without increased pre-PCI cTn, post-PCI cTn elevation is significantly predictive of adverse events (Nageh, Sherwood et al. 2003; Nageh, Sherwood et al. 2005). Thus, both pre- and post-PCI cTn level appear to be a useful prognostic biomarker under different circumstances. Patients who have undergone coronary artery bypass grafting (CABG) would also have elevated levels of cTn secondary to the procedure itself. High level of post-procedure cTn after CABG is associated with poor outcomes in these patients (Bonnefoy, Filley et al. 1998; Carrier, Pellerin et al. 2000).

Patients with chronic renal disease (CRD) often have elevated levels of cTnT in the absence of acute coronary syndrome (Freda, Tang et al. 2002; De Zoysa, 2004). This is because cTnT is renally cleared and poor renal function leads to an accumulation of cTnT. One way to determine if a CRD patient has ACS is to compare the cTnT level to the patient’s previously established baseline level if available. Other information such as patients’ age, clinical risk factors and ECG should also be used to support clinical triage decisions. However, in the absence of this critical information, one should treat the patient as an ACS patient (Aviles, Askari et al. 2002). There are many other clinical situations unrelated to cardiac ischemia in which cTN level may be elevated. These conditions include pericarditis (Bonnefoy, Godon et al. 2000; Brandt, Filzmaier et al. 2001), congestive heart failure (Missov, Calzolari et al. 1997; Horwich, Patel et al. 2003; Perna, Macin et al. 2004), drug toxicity (Cardinale, Sandri et al. 2004) and sepsis (Ammann, Maggiorini et al. 2003) for example.

In the general population, the cTnT level of some individuals is increased. This elevation may indicate subclinical cardiac injury. In a general population study, plasma cTnT levels of 3557 subjects were evaluated. cTnT elevation (≥0.01 μg/L) was found to be independently correlated with congestive heart failure, left ventricular hypertrophy, diabetes mellitus and chronic renal diseases by multivariable logistic regression analysis (Wallace, Abdullah et al. 2006). These data suggest that a subject with even a minute increase of cTnT may be at increase risk of cardiovascular events. Also, the upper limit of normal troponin level for the immunoassays should be lower than the current 0.01 μg/L in order to better detect at-risk patients.

### Development of inflammatory biomarkers for predicting upcoming cardiac events

A number of inflammatory markers have been studied for their potential as independent markers in detecting risk prior to ACS. Inflammatory markers are often elevated before the occurrence of myocardial necrosis. Thus, they may be valuable in reporting the risk level of patients who are negative for troponin or CK-MB. Patients at risk for non-ST-elevation myocardial infarction or unstable angina with negative troponin and no or atypical symptoms are perfect examples. These “at-risk” subjects in the general population who have not developed any symptom or atypical symptoms may benefit from the measurements of these markers and triaged to early preventive therapies if these markers are elevated. Some of the inflammatory markers that have been studied include interleukin-6 (IL-6), soluble CD40 ligand (sCD40L) and C-reactive protein (CRP). The elevation of IL-6 within the first two days of hospitalization in patients with ACS was associated with adverse in-hospital outcomes (Biasucci, Liuzzo et al. 1999). In a prospective study of 14,916 healthy males, an association of IL-6 levels and myocardial infarction was observed during a 6-year follow-up period. Of the participants, 202 subjects developed myocardial infarction with significantly higher level of baseline IL-6 in comparison to the matched control subjects (Ridker, Rifai et al. 2000). The risk of future myocardial infarction was also increased with increasing level of IL-6. The elevation of sCD40L is also associated with acute coronary syndrome. In a prospective study of 28,263 healthy middle-aged American women, 130 of them suffered a cardiovascular event during a 4-year follow-up. This group was found to have a modest yet statistically significant increased baseline sCD40L (2.86 ng/mL) versus the 130 age- and smoking- matched subjects (2.09 ng/mL, p < 0.02) (Schonbeck, Varo et al. 2001). Of the different inflammatory markers, CRP probably has received most of the attention. A number of studies have found that CRP is a powerful independent predictor of future cardiovascular risk (Liuzzo, Biasucci et al. 1994; Kuller, Tracy et al. 1996; Ridker, Cushman et al. 1997; Tracy, Lemaitre et al. 1997; Morrow, Rifai et al. 1998; Koenig, Sund et al. 1999; Danesh, Whincup et al. 2000; Heeschen, Hamm et al. 2000; Lindahl, Toss et al. 2000; Ridker, Hennekens et al. 2000; Lowe, Yarnell et al. 2001; Ridker, Rifai et al. 2001; Ridker, Stampfer et al. 2001; Mueller, Buettner et al. 2002). While it is a strong predictor, two recent studies however showed that the addition of CRP to traditional risk factors did not significantly improve prediction of cardiovascular disease (Cook, Buring et al. 2006; Folsom, Chambless et al. 2006). The difficulty of applying these inflammatory markers to current clinical settings is the event rates associated with elevation of these markers are low and there are no clinical trials to offer therapeutic guidance in these patients with elevated inflammatory markers. As a result, asymptomatic patients may be advised to be treated with often expensive prophalytic therapy unnecessarily. Further studies therefore will certainly be needed to demonstrate the utility of inflammatory markers as independent risk factor and how to apply them in the clinic. Perhaps, a multi-marker approach could be used to risk stratify patients.

### Development of transcriptomic and genomic markers biomarkers for identifying at-risk patient and implementing pharmacogenomic therapies

The advancement of molecular genetics has ushered in novel approaches to identify cardiovascular biomarkers. Two current molecular approaches are transcriptomic analysis and genome-wide scanning of mRNA and genes associated with the development of cardiovascular diseases. A number of promising genetic biomarkers have been identified by these techniques.

#### Platelet transcriptome

The analysis of transcriptome in platelets represents a potential novel method for identifying biomarkers for cardiovascular patients. Human platelets possess functional spliceosome, small nuclear RNAs, splicing proteins, and endogenous pre-mRNAs. Among them are pre-mRNAs for cardiovascular-related proteins. Upon platelet activation and integrin engagement, the spliceosome excise the introns in pre-mRNAs to generate the mature mRNAs for the translation of the mRNA products (Weyrich, Dixon et al. 1998; Lindemann, Tolley et al. 2001; Denis, Tolley et al. 2005). Therefore, the changes in the mRNA transcripts and their products in platelets may serve as biomarker for establishing risk factor and prognosis for cardiovascular disease. Since coronary thrombus formation and platelet activation precedes myocardial injury, platelet transcriptome activation profiling may offer an earlier diagnosis, possibly many hours before myocardial injury and troponin elevation.

By profiling the platelet mRNA from patients with STEMI, myeloid-related protein 14 (MRP-14), a protein which has been reported to modulate calcium signaling, arachidonic acid metabolism, cytoskeletal reorganization, and leukocyte trafficking (Donato, 2003; Vogl, Ludwig et al. 2004), appeared to be a novel determinant of cardiovascular events (Healy, Pickard et al. 2006). In this study, the platelet transcriptome of patients with acute ST-elevation (n = 16) was compared to patients with stable coronary artery disease (n = 44). By microarray analyses, the median expression of MRP-14 in STEMI patients was 2.2 fold higher than control patients with stable coronary artery disease (CAD). With real-time PCR, the level of MRP-14 was also 2.2 fold higher in STEMI patients. Plasma measurements further confirmed these findings. MRP-14 exists as MRP-8/14 heterodimer in plasma. STEMI patients had a plasma MRP-8/14 concentration of 17.0 μg/mL, versus 8.0 μg/mL in stable CAD patients (p < 0.001). In a subsequent prospective study with a median follow-up time of 2.9 years, subjects with MRP-8/14 plasma concentration in the highest quartile (>3.36 μg/mL) had a 3.8-fold increase in risk in developing a first cardiovascular event than the subjects in the lowest quartile (<1.15 μg/mL). In the same study, the mRNA CD69 was also found to be differentially transcripted in STEMI patients (Healy, Pickard et al. 2006). However, its potential as a biomarker in predicting cardiovascular events remained to be explored.

The study of platelet mRNA has also led to the finding of *de novo* synthesis of cycloxygenase-1 (COX-1) in activated platelets (Evangelista, Manarini et al. 2006). The activation of platelets leads to the release of arachidonic acid by phospholipase A_2_ and the formation of thromboxane A_2_ (TXA_2_) by COX-1. The release of TXA_2_ further potentiates platelet activation and aggregation. Aspirin inhibits this process by the irreversible acetylation of serine-529 on COX-1. In this platelet mRNA study, washed platelets with COX-1 activity inhibited by aspirin regained ability to synthesize TXA_2_ 24 hours after being treated with thrombin and fibrinogen (Evangelista, Manarini et al. 2006). The authors proposed that platelets overcome aspirin inhibition to generate TXA_2_ by the activation-induced *de novo* synthesis of COX-1 and hypothesized that this observation as being one of the mechanisms for aspirin resistance, especially for people on low-dose aspirin. They further supported their theory by showing that COX-1 mRNA is present in platelets and the exposure of aspirin-treated platelets to thrombin and fibrinogen led to the translation mRNA as evidenced by the incorporation of [^35^S]-methionine into the newly synthesized COX-1 protein. TXA_2_ is a potent platelet activator and only a small amount is necessary to activate platelets. In fact, it has been shown that in order to inhibit the platelet activating effect of TXA_2_, aspirin needs to inhibit over 95% of the COX-1 activity. Their hypothesis and observation may have an implication in terms of individualizing aspirin therapy in order to suppress the recovery of COX-1 activity caused by *de novo* synthesis. In a clinical study by the same group (Sciulli, Renda et al. 2006), platelets from patients experiencing CHD generated higher levels of TXB_2_ than healthy subjects even though both groups were on low-dose aspirin. Whether there is a causal relationship between *de novo* COX-1 synthesis and high levels of TXB_2_ in CHD patients remained to be elucidated. If *de novo* COX-1 synthesis is further proven by other studies to be associated with CHD, COX-1 mRNA may serve as a risk biomarker for cardiovascular patients. Of note is that, even though a number of studies have identified aspirin resistance patients whose platelets remained responsive to agonists after being treated with aspirin (Gum, Kottke-Marchant et al. 2001; Eikelboom, Hirsh et al. 2002; Gum, Kottke-Marchant et al. 2003), the mechanism behind these observations remains unclear and controversial with some investigators believing that the majority of aspirin non-responsiveness is due to patient non-compliance with taking aspirin (Cotter, Shemesh et al. 2004; Dalen, 2007). Perhaps the observation of *de novo* synthesis COX-1 represents one of the mechanisms causing aspirin resistance.

#### Single nucleotide polymorphisms (SNPs) as genetic markers implicated in myocardial infarction

After the completion of the human genome project, much research has been devoted into identifying genetic markers for predicting risk of cardiovascular disease. Genome-wide scanning for SNPs in genes implicated in coronary disease has been pursued by different groups. By scanning the microsatellite markers distributed in genomes of families and siblings, a number of studies have identified the chromosomal location of loci associated with cardiovascular disease by linkage analysis.

One of the genes that have been associated to cardiovascular disease is 5-lipoxygenase activating protein (FLAP) (Helgadottir, Manolescu et al. 2004). In this report, 296 Icelandic families with 713 individuals with myocardial infarction and 1741 individuals of their first-degree relative were analyzed. A set of 1068 microsatellites was scanned. A 4-SNP haplotype, called HapA, in the gene *ALOX5AP* on chromosome 13q12–13 for encoding FLAP was associated with a two times greater risk in myocardial infarction and stroke. In this study, another 4-SNP haplotype of *ALOX5AP*, called HapB, in the United Kingdom population was also associated with a 2 times greater risk of myocardial infarction. FLAP regulates the leukotriene biosynthetic pathway that results in the generation of leukotriene A4 (LTA4), LTB4, LTC4, LTD4, and LTE4. Among these leukotrienes, LTB4 is a potent pro-inflammatory mediator. To determine if the leukotriene pathway may be involved in myocardial infarctions, the level of LTB4 generation by neutrophils activated by ionomycin in individuals with HapA haplotype who suffered from myocardial infarction was measured. Ionomycin-induced LTB4 level was significantly higher in the MI group than the control group. Currently, a FLAP inhibitor is being developed for the prevention of thrombotic events in patients with at-risk haplotype (Hakonarson, Thorvaldsson et al. 2005). The success of this trial would demonstrate the application of pharmacogenomics and personalized medicine in the field of cardiovascular disease.

LTA4 hydrolase, another protein involved in the leukotriene pathway and encoded by the *LTAH* gene, was also found to be associated with myocardial infarction risk by genome-wide scanning (Helgadottir, Manolescu et al. 2006). HapK haplotype in the *LTAH* gene on chromosome 12 contributes a relative risk of 1.45 and 1.16 for myocardial infarction in Icelanders and European Americans respectively, whereas HapK confers a relative risk of 3.50 in African Americans. This observation suggests that the HapK haplotype probably interacts with other genetic or environmental risk factors specific to African Americans and increases the relative risk of myocardial infarction in this ethnic group. This haplotype highlights how pharmacogenomics can be applied to benefit a population subgroup. The ethnic specificity of genetic marker can help clinicians in identifying high risk patients by associating genetic biomarker with a specific subpopulation background.

A third gene that has been identified to be associated with cardiovascular disease by genome-wide scanning is myocyte enhancing factor 2A (MEF2A) on chromosome 15q26 (Wang, Fan et al. 2003). MEF2A is a member of the myocyte enhancer family of transcription factors. A 21-base pair deletion of the stop codon in exon 11 was present in all individuals with coronary artery disease in the family that was examined. The deletion of the 21 base pairs results in the removal of 7 amino acids in a region that has been demonstrated to be important for nuclear localization of the MEF2A. Another group recently has reported that subjects with a Pro279Leu variant of MEF2A in exon 7 has an odds ratio of 3.1 for myocardial infarction (Gonzalez, Garcia-Castro et al. 2006). This observation lends further support to the hypothesis that MEF2A is associated with MI.

Before the availability of genome-wide scanning, a number of genes associated with myocardial infarction have already been identified by SNPs studies. These genes include thrombospondin 4 (McCarthy, Parker et al. 2004), thrombospondin 2 (McCarthy, Parker et al. 2004), lymphotoxin-α and galectin-2. As genome-wide scanning become more common, the discovery rate of disease-causing genes will be certainly accelerated. Although this method offers a powerful and unbiased method in identifying genes implicated in cardiovascular disease, the results need to be interpreted with caution. The penetrance of cardiovascular risk confers by a particular mutation in a gene is often modulated by other genetic and environmental factors. Thus, a high risk factor in one population may not always manifest as such in another population, for example, the HapK haplotype of LTA4 hydrolase. The use of genetic biomarkers not only provides a novel way in identifying at-risk patients but has the potential of tailoring a gene-specific therapeutic regimen for these patients.

## Bioassays for Evaluating the Efficacy of Cardiovascular Therapies

Different individuals respond to antithrombotic drugs differently. Bioassays that can evaluate the efficacy of cardiovascular drug regimen in patients would ensure they are all receiving the desired therapeutic effect despite differences in their genetic makeup. This is crucial not only for determining the efficacious dose of antithrombotic drugs for an individual in an acute setting but also for prophylactic protection. Therefore, an efficacy assay which is predictive of clinical outcome should be an integral part of patient management as its impact would be more direct and immediate. Current antithrombotic therapies include the use of antiplatelet agents and anticoagulants. While these drugs have provided immense benefits for many patients, a significant number of patients received insufficient protection from these drugs, as illustrated by the SYNERGY trial. In this trial, high risk patients with acute coronary syndrome were treated with the best known combination of antithrombotic drugs. Yet 14% of the patients still experienced death or myocardial infarction within the first 30 days (Ferguson, Califf et al. 2004) and 17.6% within the first 6 months (Mahaffey, Cohen et al. 2005). With better monitoring of antithrombotic therapy, perhaps one could identify patients who would require more aggressive or alternative treatments before events occurred.

The two most commonly used antiplatelet drugs for chronic treatment are aspirin and clopidogrel. Their targets, COX-1 and P2Y12 respectively, are known to be important in platelet activation and thrombus formation. In recent studies, it has become apparent that a portion of patients are resistant to these drugs and more prone to adverse clinical events. Based on ADP- and arachidonic acid-induced platelet aggregation, 5.2% of stable patients with cardiovascular disease (n = 326) were found to be aspirin-resistant. This group had a 3-fold increase in death, myocardial infarction, or cerebrovascular accident (Gum, Kottke-Marchant et al. 2003). In another aspirin-resistance study of patients (n = 976) at high risk for cardiovascular events, patients with upper quartile serum TXB_2_ levels were more likely to suffer an MI (OR = 2) or cardiovascular death (OR = 3.5) than patients in the lower quartile (Eikelboom, Hirsh et al. 2002). Clopidogrel-resistance is also prevalent among cardiovascular patients. In one study, ADP-induced platelet aggregation, GPIIb/IIIa activation and P-selectin expression were used to evaluate the efficacy of clopidogrel. Thirty one percent and 15% of the PCI patients were non-responsive to clopidogrel therapy 5 and 30 days after PCI respectively (Gurbel, Bliden et al. 2003). In another study of PCI patients (n = 60), patients in the upper quartile of clopidogrel resistance, based on ADP-induced aggregation, have a 40% event rate within 6 months compared to none in patients who are responsive to clopidogrel (Matezky S et al. Circ, 2004 109:3171). Patients resistant to both aspirin and clopidiogrel have also been identified (Lev, Patel et al. 2006). In a group of PCI patients, 12.7% and 24% of the patients (n = 150) were found to be aspirin- and clopidogrel-resistant, respectively. Of the aspirin-resistant patients, 47.4% of them were also resistant to clopidogrel. This collection of studies highlights the frequency of aspirin- and clopidogrel-resistance in a clinical setting and how bioassays would help clinicians in identifying therapy-resistant patients who are not receiving the intended therapeutic benefit from these drugs. Currently, for the detection of aspirin-resistant patients, collagen-, ADP-, and arachidonic acid (AA)-induced platelet aggregation, Ultegra VerifyNow Aspirin assay, serum TXB_2_ level, urinary TXB_2_, and PFA-100 platelet function analyzer have been used. For the detection of clopidogrel-resistant patients, ADP-induced platelet aggregation and Ultegra VerifyNow P2Y12 assay have been utilized. However, these assays remain to be validated prospectively with clinical outcomes.

While these bioassays can evaluate the efficacy of aspirin or clopidogrel therapy, they are only effective in monitoring one specific platelet activation pathway at a time. However, current anti-thrombotic therapies involve the inhibition of multiple pathways by multiple drugs, for example, the combination of aspirin and clopidogrel. Thus, multiple assays need to be performed. This way is not only laborious and time-consuming in a clinical setting but also ineffective in providing a global physiological analysis of the antithrombotic effect of the combination therapies. Therefore, an optimal assay should be sensitive to detect all the affected thrombosis pathways concomitantly.

Thrombosis is usually initiated by the exposure of prothrombotic stimuli on the injured vessel wall or ruptured plaque to the blood flow. Platelets then accumulate at the lesion site and form a thrombus. The growth of thrombus in the coronary artery may lead to a reduction or stoppage of blood flow to cause myocardial ischemia. Aspirin and clopidogrel combination prevent occlusive thrombi not only by inhibiting platelet activation but also by promoting thrombus destabilization. Therefore, it will be clinically useful and optimal to have a bioassay for monitoring the efficacy of these drugs alone or in combination, for continuous monitoring of the thrombus formation and thrombus destabilization process, especially in patients presenting with acute myocardial infarction. The measurement of thrombus formation in a perfusion chamber coated with prothrombotic stimuli may represent an optimal bioassay (Sakariassen, Joss et al. 1990; Andre, Arbeille et al. 1996; Sakariassen, Hanson et al. 2001). By fluorescently labeling platelets, and coupling the assay to a computerized real-time monitoring system, continuous measurement of platelet adhesion to a prothrombotic surface, thrombus propagation, and thrombus stability would become feasible. The adaptation of this technique into an easy to use format may provide a new clinical assay for monitoring the efficacy of these antithrombotic drugs in patient.

## Summary

Cardiac troponin is currently the preferred biomarker for evaluating the degree of myocardial damage a patient has sustained. The use of troponin together with other diagnostic tools has allowed physicians to prescribe early proper treatment for patients with ACS. While the current cTn immunoassays are useful for diagnosing and saving patients in an acute setting, clinicians do not yet have a way to identify otherwise currently healthy subjects who would suffer from ACS in their future. Perhaps, the recent identification of genetic markers may remedy the situation. Subjects carrying the susceptible haplotypes could be identified years before any cardiovascular disease develops. The successful validation of these markers would allow the practice of personalized pharmacogenomics in the cardiovascular disease area. One day, physicians might be able to prescribe personalized disease-prevention program based on a person’s genome. In the future, with new genetic tools for screening high-risk patients, acute biomarkers for determining myocardial damage and novel bioassays for monitoring drug efficacy, we will be able to better identify, monitor, and treat patients with acute coronary syndrome.
